# 

**Published:** 1918-07-06

**Authors:** 


### CONTENTS.

| | PAGE |
|---|---|
| **United and Joyous** ... ... ... ... ... ... | 295 |
| **The Men of Fifty** ... ... ... ... ... ... | 296 |
| **Hospital and Institutional News** ... ... ... | 297 |
| Death of a Hospital Chairman—Inter-Allied Exhibition of After-care Methods—The Films and the Welsh Section—The Stock Exchange and the Red Cross—Nomenclature of Hospitals: An Odd Omission—Venereal Diseases: A Meeting in Edinburgh—Proposed Memorial to a Hospital Chairman—New Secretary at Chichester—Hospital Demonstrators and Military Service—Serious Fire at a V.A.D. Hospital—The "Failure" of Sanatoria—Some Misconceptions Exposed—The Guardians of the Milk Supply—The "Waac," the "Wren," and the Mental Nurse—Sanitary Science in South Wales—The Imbecile Attendant—Simple but Expedient—Board Wages for Sisters on Holidays—Municipal Dentistry—This Week's Drug Market—To Our Readers. | |
| **On Breathlessness in Cardiac Disease** ... | 301 |
| The "Effort Syndrome" in Relation to Blood Quality. | |
| **A Piece of Useful Reconstruction** ... ... | 302 |
| Census of Limbless Civilians in Wales. | |
| **The Institutional Library** ... ... ... ... | 305 |
| **Temporary Artificial Legs** ... ... ... ... | 303 |
| As Made at the Pavilion General Hospital, Brighton. | |
| **The Editor's Letter-Box** ... ... ... ... | 307 |
| The Training of an Asylum Matron. | |
| **A New Charter of Liberty** ... ... ... ... | 308 |
| "Ierne" and Nurses All. | |
| **The Matrons' and Sisters' Department** ... | 309 |
| Training-Schools and Public Health Work. | |
| Round the Hospitals. | |
| Royal National Pension Fund's 6th Quinquennium—Overseas Nursing Association not C.N.A.—The College Vigorous at Derby—The Asylum Matron's Training—The New Food Orders—Edinburgh Royal Infirmary Nurses. | |
| **Parliament and the Profession** ... ... ... | 313 |
| **A Village Committee** ... ... ... ... ... | 313 |
| **A Display in Domestic Economy** ... ... | 313 |
| **Matrons' and Sisters' Appointments** ... .. | 314 |
| **The Book Market** ... ... ... ... ... ... | 314 |
| **Recent Official Publications** ... ... ... | 314 |
| **Lectures on Venereal Disease** ... ... ... | 314 |
| **The Institutional Worker** ... .. | (Suppl.) 1 |

